# Impact of Puberty in Girls on Prevalence of Primary Headache Disorder Among Female Schoolchildren in Kuwait

**DOI:** 10.3389/fneur.2020.00594

**Published:** 2020-07-17

**Authors:** Abdelrahman Alashqar, Sameera Shuaibi, Samar Farouk Ahmed, Hawraa AlThufairi, Shaikhah Owayed, Fajer AlHamdan, Raed Alroughani, Jasem Yousef Al-Hashel

**Affiliations:** ^1^Obstetrics and Gynecology Department, Ministry of Health, Kuwait City, Kuwait; ^2^Internal Medicine Department, Ministry of Health, Kuwait City, Kuwait; ^3^Neurology Department, Ibn Sina Hospital, Safat, Kuwait; ^4^Neuropsychiatry Department, Faculty of Medicine, Al-Minia University, Minia, Egypt; ^5^Faculty of Medicine, Kuwait University, Safat, Kuwait; ^6^Division of Neurology, Department of Medicine, Amiri Hospital, Sharq, Kuwait

**Keywords:** migraine, tension type headache, primary headache, puberty, school students, sex hormones

## Abstract

**Background:** The prevalence of primary headaches in the pediatric population is shaped by many factors, of which pubertal status may possibly play a substantial role. Epidemiological studies in the pediatric population in the gulf region remain scarce.

**Aims and objectives:** To examine the impact of puberty on the prevalence of primary headache disorders among female schoolchildren in Kuwait.

**Methods:** We conducted a cross-sectional study that included Kuwaiti primary and middle schoolgirls in randomly selected schools located in two governorates in Kuwait during the academic year 2018/2019. Prevalence of headache was assessed using the Headache-Attributed Restriction, Disability, Social Handicap and Impaired Participation (HARDSHIP) questionnaire for children and adolescents. Female students were asked about their menarchal status and whether they attained menarche before or after experiencing headaches.

**Results:** The questionnaire was completed by 669 girls with a mean age of 11.44 ± 2.14 years. The 1-year prevalence of migraine headache disorder among girls was 23.62%, and the lifetime prevalence of any headache was 84.9%, whereas the 1-year prevalence of primary headache disorders was 47.98%. The mean age of girls with headaches was 11.44 ± 2.14 years. With respect to diagnostic criteria, migraine headache was the most frequently reported (23.62%), followed by tension-type headaches (20.93%), chronic headaches (2.99%), and probable medication-overuse headaches (0.45%). Postpubertal females were at significantly higher risk of having primary headaches compared to their prepubertal counterparts (64.26 vs. 34%; *p* < 0.0001). All types of primary headaches were more significantly prevalent among postpubertal girls compared to those who are prepubertal.

**Conclusion:** Migraine headache is commonly reported among Kuwaiti schoolgirls. Postpubertal females are at higher risk of developing primary headaches compared to prepubertal females. Pubertal transition and female sex hormones may play a significant role in the pathophysiology of headaches, migraines in particular, and further research is therefore needed to investigate the underlying mechanisms.

## Introduction

Headache is one of the most common disorders in childhood, with an estimated 75% of the pediatric population reporting a significant headache by 15 years of age (1). It is defined as head pain that occurs anywhere in the head or neck areas (2). Headaches among children and adolescents can be categorized into primary and secondary headaches; the most common primary headaches constitute migraine headaches, tension-type headaches, and cluster headaches and are similarly categorized in adults. Pediatric headaches are reportedly more common among older children; they are of rare occurrence prior to 4 years of age, and their incidence peaks at the age of 13 (3, 4). Differential risk of developing primary headaches between both genders has been already established in the literature. Migraines, for example, are two to three times more common in females than in males (5). When addressing pediatric migraine, rates tend to be similar between boys and girls before 10 years of age; however, as approaching adolescence, girls are reportedly at higher risk of developing migraines (6). This sex-specific prevalence suggests a role for sex hormones on the course of primary headaches in both genders (7). In addition, these observations postulate a substantial role for puberty and its associated hormonal transition in increasing an adolescent female's risk of primary headaches, migraine in particular. In fact, migraine headaches are known to share a close association with menstruation, further supporting this hormonal hypothesis. Postpubertal modifications in certain brain areas, specifically the hypothalamus, were thought to act as major modulators of this risk (8). Nevertheless, accurate mechanisms remain not fully understood and exact culprits are to be further delineated. Studies addressing the prevalence of headaches and their association with puberty are scarce worldwide among the pediatric and adolescent age groups; in particular, the Arab region, including Kuwait, are falling short with this regard and thus, studies of this kind are a necessity. We reported in our previous work that the 1-year primary headache prevalence did not significantly differ between males and females in the age group 6–11 years, while it was significantly higher in females compared to males in the age group 12–17 years. We, therefore, attempt in our study to address the impact of puberty on increasing the prevalence of migraine headache among adolescent girls.

## Methods

This is a cross-sectional study with a school-based sample whereby questionnaires were distributed to primary and middle schoolgirls in governmental schools in Kuwait. Female schoolchildren were randomly selected from two major governorates in Kuwait: Al-Farwaniyah, the most densely populated and farther away from the center of the State of Kuwait, and Hawally, which is more urbanized and central in location. These two governorates were chosen to cover the geographic diversity of Kuwait. We included Kuwaiti schoolgirls aged 7–16 years. Based on the information from the Kuwait Ministry of Education in academic year 2018/2019, the number of Kuwaiti girls in primary and middle schools was 71,448. The sample size was calculated and determined to be 550 using a special formula based on the prevalence of headache reported from previous national and international epidemiological studies, which was around 54.4% for headaches and 9.1% for migraine [9–10]. The sample was increased by 20% to overcome non-response rates and missing data. The questionnaires were distributed to 900 girls, of which 669 completed the questionnaires and were included in the analysis. A total of 231 questionnaires were not analyzed because 110 girls refused to participate while the rest of questionnaires were incomplete. A representative random sample of female schools was selected and thereafter stratified by grade (3rd, 5th, 7th, 9th) to cover the age spectrum from childhood through adolescence. All children and adolescents within these selected classes were included, except for those who refused to participate, were non-Kuwaiti nationals, have history of a medical or neurological disease, or were absent on the day of the survey. Questionnaire distribution and data collection were organized and conducted during a school class as a paper–pencil version. To collect the study data, well-trained physicians conducted face-to-face interviews using the Child HARDSHIP questionnaire for children aged 6–11 years and Adolescent HARDSHIP questionnaire for adolescents aged 12–17 years (9). A diagnosis of headache was made by using the HARDSHIP algorithm (10). Subjects who were diagnosed with migraine by the HARDSHIP questionnaire; their diagnosis was confirmed by a headache specialist by applying ICHD-III (11). Girls answered the question: “Have you reached your menarche?” with either a yes or a no. Girls who attained their menarche were further asked whether they had their first menstruation before or after their headache started. If menarche was reached before experiencing headaches, the participant was included in the postpubertal group. The Ministry of Health and Ministry of Education in Kuwait approved the study. Participants were given a simple explanation about the aim of the study being considered an ethical issue. Verbal and written informed consents were obtained from all participants and their parents on the day before the questionnaires were distributed. The participants were granted the right to decline participation at any time during data collection. All data were protected in accordance with the ethical guidelines of the Council for International Organizations of Medical Sciences (12, 13).

### Statistical Analysis

The data from completed questionnaire were entered on IBM SPSS Statistics 20.0. Entries were double-checked with inconsistencies reconciled by reference to the original documents. An error rate of 1.9% was identified. One-year prevalence of primary headache disorders was also calculated as percentages with 95% confidence intervals (CIs). We used proportions, 95% CIs, means, and standard deviations (SDs) to summarize the distribution of variables as well as chi-square for significance of differences. *P* < 0.05 was considered statistically significant.

## Results

Our cohort included 669 girls who completed the questionnaire. The mean age of our female participants was 11.44 ± 2.14 years (Table 1). The lifetime prevalence of any headache was reported to be 84.9%, while the 1-year prevalence of primary headaches was 47.98%. After stratifying primary headaches, migraines were the most frequently reported by female students (23.62%) followed by tension-type headaches (20.93%). The least experienced primary headache among females was medication-overuse headache (0.45%).

**Table 1:** Demographic data and prevalence of headache among female school students (*N* = 669).

**Variables**	**Total female school students**
	**M ±*SD*/no. (%) [CI 95%]**
Mean age in years	11.44 ± 2.14
Range	7–16
Life time prevalence of any headache	568 (84.90%) [81.98–87.42]
1-year prevalence of any headache	524 (78.33%) [75.04–81.29]
1-year prevalence of primary headache disorder	321 (47.98%) [44.22–51.77
Migraine	158 (23.62%) [20.55–26.98]
TTH	140 (20.93%) [18.01–24.18]
CH	20 (2.99%) [1.92–4.60]
pMOH	3 (0.45%) [0.09–1.38]

*TTH, tension-type headache; CH, chronic headache; pMOH, probable medication-overuse headache; M, mean, SD, standard deviation; CI 95%, confidence interval 95%*.

Female schoolers were stratified according to their pubertal status (Table 2). The 1-year prevalence of primary headaches was significantly associated with pubertal status among females (*P* < 0.0001) with more pubertal females (64.26%) reporting a primary headache compared to prepubertal females (34.18%). Prevalence of primary headaches in prepubertal girls vs. postpubertal girls did not show a significant difference (33.3 vs. 33.1%, *p* < 0.986) among primary schoolgirls; however, among middle schoolers, the difference was rendered significant (48.6 vs. 12.8%, *p* < 0.010). Among prepubertal females, tension-type headache was the most frequent type (17.35%), whereas pubertal females reported more migraine headaches (33.57%) than any other type. All types of primary headaches were more prevalent among pubertal females with migraine (*P* < 0.0001); tension-type headaches showing a significant association with pubertal status (*P* < 0.007). Table 3 displays the 1-year prevalence of types of primary headache disorders stratified by age and gender. There was significant difference between prevalence of primary headache disorders at older ages but no significant differences at young age.

**Table 2:** One-year prevalence of types of primary headache disorders stratified by puberty among female school students (*N* = 669).

**Types of primary headache disorder**	**Prepubertal (*N* = 392)**	**Postpubertal (*N* = 277)**	***P*-value**
	***N* (%) [CI 95%]**	***N* (%) [CI 95%]**	
Primary headache disorder	134 (34.18%)	178 (64.26%)	0.0001^*^
	[29.66–39.01]	[58.45–69.68]	
Migraine	65 (16.58%)	93 (33. 57%)	0.0001^*^
	[13.21–20.60]	[28.27–39.33]	
TTH	68 (17.35%)	72 (25.99%)	0.007^*^
	[13.91–21.42]	[21.17–31.47]	
CH	8 (2.04%)	12 (4.33%)	0.087
	[0.97–4.05]	[2.42–7.50]	
pMOH	2 (0.51%)	1 (0.36%)	0.776
	[0.01–1.97]	[0.01–2.23]	

*TTH, tension-type headache; CH, chronic headache; pMOH, probable medication-overuse headache; M, mean, SD, standard deviation; CI 95%, confidence interval 95%*.

* *Indicates significant*.

**Table 3:** One-year prevalence of primary headache disorders stratified by age and puberty (*N* = 669).

**Variable**	**Age 7–10 years**		**11 years**		**12 years**		**13 years**		**14–17 years**	
	**(*****N*** **=** **311)**		***N*** **=** **(47)**		***N*** **=** **(114)**		***N*** **=(52)**		***N*** **=** **(145)**	
	**Total**	**Headache cases**.	**Total**	**Headache cases**.	**Total**	**Headache cases**.	**Total**	**Headache cases**.	**Total**	**Headache cases**.
	**number**	**Number (%)**	**number**	**Number (%)**	**number**	**Number (%)**	**number**	**Number (%)**	**number**	**Number (%)**
Prepubertal prevalence of primary headache	297	99 (33.3)	34	13 (38.2)	49	21 (42.9)	7	0	5	0
Postpubertal prevalence of primary headache	14	6 (42.9)	13	9 (69.2)	65	40 (61.5)	45	32 (71.7)	140	101 (72.1)
*P*-value	0.461		0.057		0.048		0.001		0.002	

## Discussion

In our study, the remarkable prevalence of primary headaches happens to be in agreement with previous studies. Philipp et al. reported that female gender was indeed associated with an increased probability of headache (14), a finding that was also reiterated by Genizi et al. who showed that girls reported a higher prevalence of headaches as well (15). Girls have consistently been reported to suffer more than boys from any headache, including migraines (16, 17). In fact, female gender was found to be closely associated with a higher prevalence of any headache, migraines, and chronic headaches and that females tend to have a higher headache prevalence with increasing age (14).

Our study showed that the 1-year prevalence of primary headaches was significantly higher in postpubertal girls compared to their prepubertal counterparts (*P* < 0.0001). A previous study demonstrated that the risk of recurrent headaches is higher in menarchal girls when compared to those without menarche (18). This intriguingly suggests hormonal changes at the time of puberty that may contribute to the development of headaches. Female hormones, estrogen in particular, modulate neuronal circuits and induce permanent molecular and morphological changes in the adolescent brain, promoting an increased tendency for headache development in females during their pubertal transition (8). Female sex hormones can interestingly cross the blood–brain barrier and exert various effects on the brain (19). Furthermore, the serotonergic system, which plays a significant role in the pathogenesis of migraine, is greatly modulated by estrogen. The latter enhances tryptophan hydroxylase expression while suppressing that of serotonin reuptake transporter (20).

After diagnostic stratification, this statistically significant difference persisted for migraine (*P* < 0.0001) and tension-type (*P* < 0.007) headaches, reiterating studies that documented an increased prevalence of primary headaches with increasing age from childhood through adolescence (21, 22). Bianchin et al. indeed reported that headache rates in girls rise linearly with increasing gynecological age (23). This remarkable increase in rates of headache subtypes between preadolescent and postpubertal females implies a potential role for puberty in migraine pathophysiology mediated by hormonal factors that parallel that transition. In fact, early menarche has been shown to be a risk factor for migraine development (24). The interplay between puberty and migraine has been thought to be mediated by changes in the hypothalamic physiology. These hypothalamic changes have been postulated to be greatly influenced by sex hormones (8). Resetting the neuroendocrine circuits in the hypothalamus can potentially impact the biology of the trigeminovascular system, the major pathway in migraine pathophysiology. Of importance, the trigeminal nucleus possesses estrogen receptors (25) and sensitization of trigeminal neurons by estradiol may be modulated through mediators such calcitonin-gene-related peptide (26), further supporting the hormonal basis of migraine pathobiology. Estradiol biosynthesis takes place in various regions of the brain, including the hypothalamus, brainstem, and basal forebrain. Many of these regions are simultaneously involved in migraine headaches (27). In addition, serum levels of estrogen and progesterone were found to be significantly higher among migraine patients throughout the menstrual cycle compared to controls (28). On the contrary, migraine prevalence tends to decrease in postmenopausal females paralleling the decline in estrogen levels during menopausal transition (8). In addition, puberty is associated with an increase in body weight, which in turn can increase the risk of migraine and possibly explain the high prevalence of migraine after puberty (29). Despite being less studied, the relationship between puberty and tension-type headache has been similarly reported in the literature. Menarchal girls have a higher tendency to develop tension-type headaches than their premenarchal counterparts. This is demonstrated by the increased incidence ratio of tension-type headache between boys and girls from 1.3:1 prepubertally to 1:1.2 after menarche (30). The mean age of our cohort was 11 years. It was reported in the literature that headaches, including migraines, have a higher occurrence in adolescents and adult females with a history of early menarche. Early menarche may possibly render females more susceptible to headaches or can be a consequence, along with migraines, of increased estrogen sensitivity (24).

### Strengths and Limitations of the Study

This study is the first of its kind in Kuwait and the Gulf region providing valuable data on the impact of pubertal status in girls on the prevalence of primary headache disorders among female schoolchildren. It generated a large randomized stratified sample representative of Kuwait. Results of this study may potentially help in predicting the future trends of primary headaches as well as deciding on the optimal management of this disorder in adolescent girls.

Our study has limitations including recall bias introduced by answering the questionnaire, and the reliance on self-reports of clinical parameters of headaches. Being a cross-sectional study, associations between headache and pubertal status may not be interpreted in a causal manner.

## Conclusions

The lifetime prevalence of any headache in female schoolchildren was 84.9%, whereas the 1-year prevalence of primary headache disorders was 47.98%. Migraine headache was the most frequently reported (23.62%), followed by tension-type headaches (20.93%). All types of primary headaches were more significantly prevalent among postpubertal girls compared to those who are prepubertal. Hence, the pubertal transition and its paralleling hormonal changes may play a potential key role in priming the female brain and its associated structures with an increased risk of headaches. Future research should target the extent to which puberty plays a pathophysiological role in the development of primary headaches, including the underlying mechanistic basis and molecular events.

## Data Availability Statement

The raw data supporting the conclusions of this article will be made available by the authors, without undue reservation.

## Ethics Statement

This study was carried out in accordance with the ethical guidelines of Kuwait Ministry of Health. The protocol was approved by the Ethics Committee of Ministry of Health. All subjects and their parents gave written informed consent in accordance with the Declaration of Helsinki.

## Author Contributions

JA-H designed the study and reviewed the manuscript. SA designed the study, performed statistical analysis, and criticized and reviewed the manuscript. AA, SS, HA, SO, and FA performed data collection and data entry and drafted the manuscript. RA reviewed the manuscript. All authors read and approved the final manuscript.

## Conflict of Interest

The authors declare that the research was conducted in the absence of any commercial or financial relationships that could be construed as a potential conflict of interest.

## Abbreviations

CH: Chronic headache
HARDSHIP, Headache-Attributed Restriction, Disability: Social Handicap and Impaired Participation
ICHD-III: International Classification of Headache Disorders III
pMOH: Probable medication-overuse headache
TTH: Tension-type headache.
